# Energy-Information Trade-Offs between Movement and Sensing

**DOI:** 10.1371/journal.pcbi.1000769

**Published:** 2010-05-06

**Authors:** Malcolm A. MacIver, Neelesh A. Patankar, Anup A. Shirgaonkar

**Affiliations:** 1 Department of Mechanical Engineering, Northwestern University, Evanston, Illinois, United States of America; 2 Department of Neurobiology and Physiology, Northwestern University, Evanston, Illinois, United States of America; 3 Department of Biomedical Engineering, Northwestern University, Evanston, Illinois, United States of America; 4 Department of Mechanical Engineering, Massachusetts Institute of Technology, Cambridge, Massachusetts, United States of America; University College London, United Kingdom

## Abstract

While there is accumulating evidence for the importance of the metabolic cost of information in sensory systems, how these costs are traded-off with movement when sensing is closely linked to movement is poorly understood. For example, if an animal needs to search a given amount of space beyond the range of its vision system, is it better to evolve a higher acuity visual system, or evolve a body movement system that can more rapidly move the body over that space? How is this trade-off dependent upon the three-dimensional shape of the field of sensory sensitivity (hereafter, sensorium)? How is it dependent upon sensorium mobility, either through rotation of the sensorium via muscles at the base of the sense organ (e.g., eye or pinna muscles) or neck rotation, or by whole body movement through space? Here we show that in an aquatic model system, the electric fish, a choice to swim in a more inefficient manner during prey search results in a higher prey encounter rate due to better sensory performance. The increase in prey encounter rate more than counterbalances the additional energy expended in swimming inefficiently. The reduction of swimming efficiency for improved sensing arises because positioning the sensory receptor surface to scan more space per unit time results in an increase in the area of the body pushing through the fluid, increasing wasteful body drag forces. We show that the improvement in sensory performance that occurs with the costly repositioning of the body depends upon having an elongated sensorium shape. Finally, we show that if the fish was able to reorient their sensorium independent of body movement, as fish with movable eyes can, there would be significant energy savings. This provides insight into the ubiquity of sensory organ mobility in animal design. This study exposes important links between the morphology of the sensorium, sensorium mobility, and behavioral strategy for maximally extracting energy from the environment. An “infomechanical” approach to complex behavior helps to elucidate how animals distribute functions across sensory systems and movement systems with their diverse energy loads.

## Author Summary

Animals thrive by sensing their environment and using the information they've gathered to guide their movement. But collecting better information can result in less efficient movement: Bicycling while standing up on the pedals may help you see over obstacles ahead of you, but it causes more air drag, forcing your legs to work harder. Nocturnal weakly electric fish search for prey with their body tilted. This tilting more than doubles the resistance to movement from the water, but because the fish's ability to sense prey improves when tilted, it is better to swim this way. Beyond a certain amount of tilt, the costs of movement become too great. Interestingly, the benefit of tilting is dependent on the shape of the volume around the fish where it detects prey. We also found that if the fish was able to swivel its region of prey sensitivity, like a vision-based animal can shift its gaze, it would save energy. This conclusion helps us understand why animals like us can move our eyes. A Polish folk saying succinctly captures the gist: “He who doesn't have it in the head has it in the legs” (Ten kto nie ma w głowie ma w nogach).

## Introduction

Animals must constantly negotiate trade-offs in sensory and motor performance. The most well known of these trade-offs occur within either movement or sensory systems, rather than between them. As an example within motor systems, fish body shapes and styles of movement that maximize cruising efficiency may suffer from poor maneuverability [1]–[3]. In sensory systems, converging signals from large numbers of photoreceptors for increased sensitivity results in reduced spatial resolution.

What about trade-offs *between* movement and sensory systems? For example, for a fixed amount of available energy from food sources, is it better to expend that energy on a larger visual sensing range (via a larger eye and the brain tissue to process signals), or to move the body more so that the effective area that is scanned is similar? One challenge in assessing such trade-offs is that it is difficult to compare measures of movement performance, such as energy efficiency, to sensory performance, such as acuity. Ultimately, however, these different subsystem performance measures translate into net energy gains and losses for the animal [4]. Consequently, examining energy provides a lens through which to look at how an animal can best trade off movement and sensing. Given that neuronal tissue requires about 20 times more energy than skeletal muscle per unit mass in mammals, where it has been measured ([5], after [6]), we already know that brains and sensory systems are metabolically expensive compared to movement systems. Recent studies have shown the important influence of the energetic costs of sensory systems, such as the role of these costs in the evolution of sensory systems (review: [7]). Although looking at energetics enables comparison of the costs of movement and sensing in behaviors where these are closely interrelated, such an analysis has rarely been performed [7].

One simple source of trade-offs between movement and sensing can be easily understood. A key role of a sensory system is to support scanning the environment for food, threats, mates, competitors, or anything else which may affect the animal's continued existence. But the space where these items of interest exist will typically exceed the range of the sensory system. To scan a larger volume of space, an animal can move its body, or evolve increased sensory range. Either approach has its associated costs. In the case of body movement, it is the cost of locomotion. The amount of locomotion needed will depend on the range of the sensory system being used, with less movement needed by long-range systems, such as vision, and more movement needed for short-range systems, such as touch. If, for example, you need to detect the location of a split on a wood table, you can use your visual system and glance at the entire surface at once (little dependence on movement), or you can move your hands across the surface and use your sense of touch to detect the split (maximal dependence on movement). In the case of evolving increased sensory range, the associated costs include more neuronal tissue, development costs, maintenance, and the cost to carry the weight of the sensory system (not insignificant for flying animals: the fly uses 3% of its energy simply to keep its visual system aloft [8]).

The above type of trade-off between movement and sensing is indirect because the problem is how best to expend a fixed amount of energy (more on movement, and less on sensing, or vice versa)— but not a case where improvement in one domain comes at the expense of performance in the other domain. An example of a more direct trade-off like this is how moving the eye faster to increase the speed of visually inspecting an area of space can directly conflict with visual performance. The conflict arises when an image passes over more than one photoreceptor acceptance angle per response time, since this results in the visual percept being degraded by motion blur [9].

A thought experiment can help expose another direct way in which a trade-off between movement and sensing can occur, one similar to the kind at issue in this study. As the effective range of a given sensory epithelium approaches zero (contact sensing), to increase the amount of space that is scanned while moving through space (for example, in a straight line) can require reorienting the sensory epithelium in a way that results in less efficient movement. For example, imagine your finger was an autonomous organism. Suppose this finger is feeling its way along a novel surface in a water current (or a stiff wind), with the long axis of the finger parallel to the direction of movement so as to minimize drag effects. Now, the back portion of the finger is scanning the same surface as was already scanned by the front. To increase the amount of space being scanned per unit time, the sensory epithelium needs to be reoriented. Ideally, the finger would be oriented perpendicular to the line of travel. This way the rate of surface scanning is maximized; but now there is also maximal projected area in the direction of travel, and thus maximal drag.

Contrast this situation with that involving a sensory epithelium whose range is far from zero, such as the retina of an eagle flying high and looking for prey on the ground. Now, suppose that the eagle is looking straight downward. The eagle's visual sensorium can be idealized as a cone whose apex is the eagle's head. The area scanned per unit time will be the width of the cone times the velocity of the eagle. If instead of looking straight down, the eagle sweeps its conical sensorium from side to side by moving its eyes, it will greatly increase the area scanned per unit time. In this case, however, to reorient the sensory epithelium through eye rotation comes at no change in the projected area of the body in the direction of travel, and thus no added costs due to increased drag. If the eyes were not movable, the eagle would have to turn its head, which could result in more drag; if the eye and head were not movable, the whole body would need to be reoriented, incurring even more costs. Note, however, that having the ability to reorient the sensory epithelium without changing body orientation can incur significant neuronal processing costs, since it may require coordinate transformations from a sensory organ-fixed coordinate frame (e.g., retinotopic coordinates) to body-fixed coordinates.

With sensors distributed over a sensory epithelium consisting of the entire body surface, as occurs in somatosensory and electrosensory systems, it becomes progressively less possible to reorient the sensory epithelium independently of full body reorientation. For example, it conflicts with the strategy of concentrating the sensors on one portion of the body which is moved with muscles, as with some eyes and pinnae. Full body reorientation, however, can be quite costly if the relative velocity between the body and the surrounding environment is sufficient to produce drag forces on the body — for example, if the animal is moving rapidly through the air.

An example of this type of trade-off between sensing and movement can be found in chemosensory behavior of the blue crab [10]. Blue crabs move sideways up-current, with their body slightly rotated into the flow. The slight rotation into the flow is believed to result in improved sensing of the local gradient of odorant molecules, as this rotation causes their primary chemosensory appendages for this behavior—their legs [11] —to be sensing slightly across the flow. Without this slight rotation, the downstream legs receive fluid in which the odorant has been mixed and diluted from hitting the upstream legs, compromising the ability to detect and localize the odorant. With the body rotated into the flow, the crab avoids this dilution and can use bilateral comparisons between chemosensory input along the legs to help guide the body to the source. However, turning the body into the flow also increases drag. As Weissburg and coworkers increased flow speed in their experimental apparatus, they found a speed at which the crab chose not to rotate the body into the flow. The cost of movement at the increased drag appears to outweigh the gain in sensory performance at this critical flow speed.

**Figure 1:**
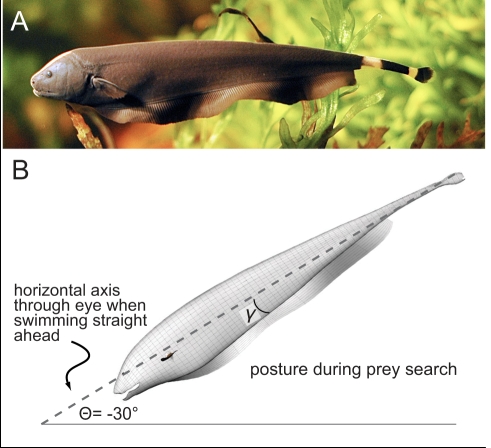
*Apteronotus albifrons*, the black ghost knifefish of South America. (A) Photograph courtesy of Per Erik Sviland. (B) Posture of fish while swimming forward in search of prey, with body pitch angle 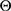 typically 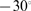 [16]. The angle 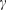 is the fin base insertion angle, typically approximately 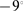 in this species.

Here we present an analysis of a conflict between efficient movement and sensory performance using the model system of weakly electric fish (Figure 1A), a leading system for the analysis of sensory function in vertebrates. These fish hunt for small insect prey at night in rivers of the Amazon Basin, through the use of an active electrosensory system. The fish generate an oscillating electric field (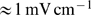 near the body), that surrounds the whole animal. When prey enter the fish's electric field, a small change in voltage occurs across the skin (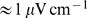) [12], [13]. This change in voltage is detected by electroreceptors covering the entire body surface. These voltage modulations are then transformed into changes in the firing rate of primary electrosensory afferents that terminate in the hind brain of the animal for further processing (reviews: [14], [15]).

While searching for prey, these fish were previously shown to hold their body with the head down at a 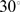 pitch while searching for prey [16], as illustrated in Figure 1B. We show that this posture significantly increases the cost of movement. However, this increased cost is more than offset by the increase in sensory performance resulting from the posture. We observed that this increase in sensory performance is dependent upon the fish having an elongated sensorium. When we examined the effect of the fish having a non-elongated sensorium, such as a blunt-shaped sensorium or a forwardly-directed visual sensorium similar in aspect ratio to a visually-guided aquatic predator, we found that there was no benefit to increasing the pitch of the body. We show that if the black ghost could swivel its sensorium independently of body movement, as visually-guided animals can swivel their sensoria, the fish would obtain a significant benefit through reduced energy expenditure for prey search.

## Results

### Resistance to Movement from the Water while Searching for Prey

Body movement through any medium results in lost energy due to friction between the medium and the body. In air these effects are slight except for flying animals. In water, with 1,000 times the density of air, these effects are significant even at relatively low speeds. As mentioned above, black ghost knifefish tilt their body while searching for prey. To estimate the energetic consequences of tilting their body from neutral (horizontal) body pitch to the measured 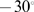, the force needed to overcome the resistance to movement (drag) through water needs to be estimated at different body pitches and movement speeds. The energy needed to overcome this resistance is then simply this force times the distance moved.

**Figure 2:**
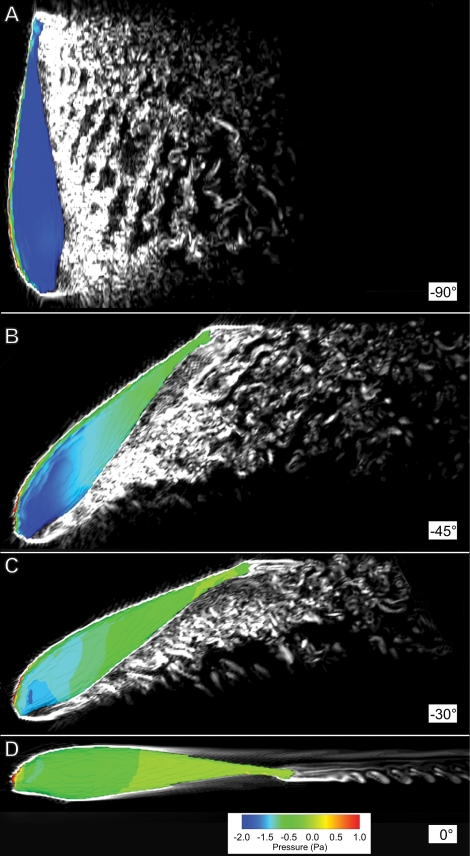
Computed wakes of a model of the black ghost at different pitch angles, at a velocity of 15 cm/s. The body is shown colored by the surface pressure deviation with respect to the hydrostatic pressure. Vorticity contours are shown in gray scale in the mid-sagittal plane of the fish. Wakes of the body at pitch angles of (A) 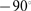; (B) 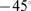; (C) 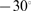; (D) 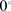.

We estimated the drag in two ways. First, we performed high resolution computational fluid dynamic simulations of the black ghost as it was being virtually towed through water. The forces on the body are easily recovered from the simulations, as are the flow patterns, which give insight into the basis of the drag forces corresponding to each body pitch angle. The computed flow patterns are shown in Figure 2. Second, we towed an accurate urethane cast of the knifefish through a large water tank at constant, behaviorally relevant velocities, measuring the steady-state resistance to movement with a force sensor that the cast was attached to.

**Figure 3:**
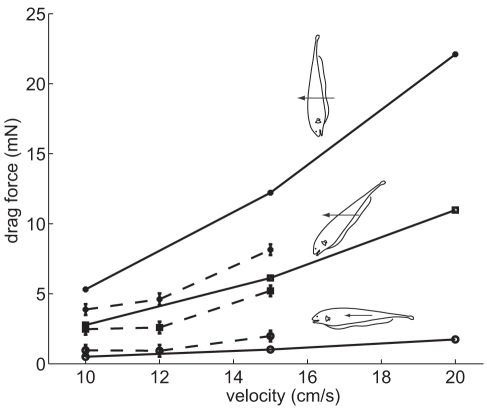
Measured and computed drag on the fish body at different body pitch angles. –
–, 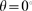 –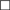
–; 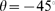; –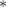
–; 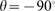. Dashed lines indicate experimentally measured drag, while solid lines show the drag estimated with computational fluid dynamics. Insets show orientation of fish cast while being towed at these angles.

We highlight results for 15 cm/s, because our prior prey capture study with the black ghost knifefish found search velocities of 9.3
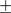
4.3 cm/s (mean and std) [16]. In that study, the tank in which we made our observations had to be small due to imaging constraints, making 15 cm/s a reasonable choice to focus on here. The drag force results are shown in Figure 3. At 15 cm/s, the measured drag force was 2.0
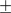
0.4 mN (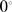), 5.2
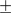
0.4 mN (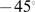), and 8.1
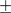
0.5 mN (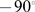). The corresponding computed drag forces were 1.0, 6.1, and 12.2 mN.

The measured drag was typically lower than the computationally estimated drag. As shown in the snapshots of the computed flow patterns around the fish being virtually towed at 15 cm/s in Figure 2, at 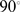 the flow separation is higher than in the other cases. Because of this degree of separation, computational fluid dynamic simulations that incorporate the effect of turbulence may be required to fully resolve the flows around the body. If turbulence is present in the empirical experiments with the fish cast, this could potentially reduce the drag. Given the disparities between measured and computed drag forces, we use the measured drag forces for the remainder of the study. Our key result, that observed pitch angles during search behavior are consistent with minimizing costs, are not affected by this choice.

### How Search Rate Changes with Body Pitch

**Figure 4:**
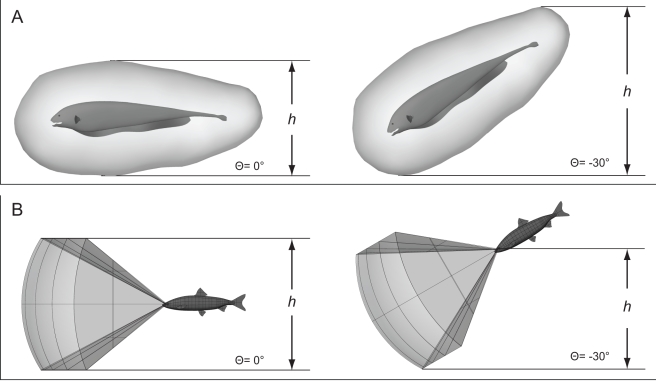
How search volume changes with body pitch. (A) Electrosensory case. A black ghost knifefish is shown with the sensorium for detecting 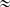
3 mm long water fleas (*Daphnia magna*). Prey anywhere on or within the surface are detectable by the fish. From [12]. The volume of water which is scanned for prey will be the fish's velocity times its duration of movement, times the projected area of the sensorium in the direction of travel. In this case, the projected area is the height *h* times the width (dimension out of the plane of the figure) of the sensorium. As the body pitch increases, *h* increases and so does the projected area. (B) Visual case, assuming no swiveling of the eyes to compensate for body pitch. A stone moroko is shown with the sensorium for detecting 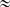
2 mm long water fleas (*Daphnia pulex*). From [22], as visualized in [12].

**Figure 5:**
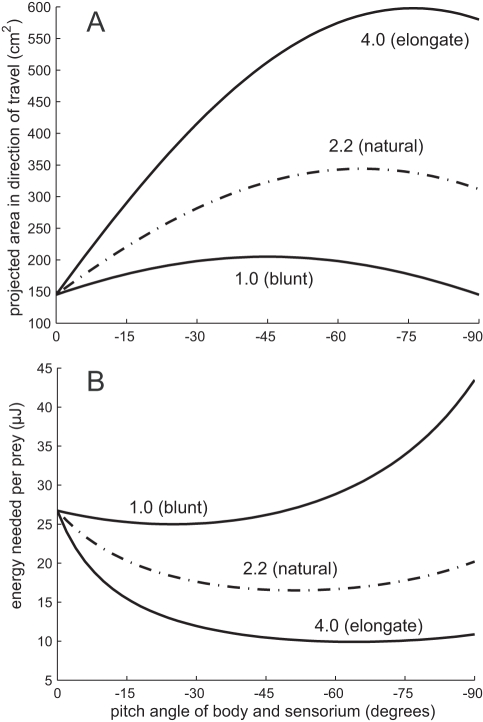
How the projected sensorium area and the energy needed to encounter one prey vary with body pitch angle and elongation factor. In each case, the number on the curve indicates the ratio of the length of the sensorium to its height. The natural case is that the sensorium is 2.2 times longer than its height. (A) Projected sensorium area in the direction of travel. (B) Energy needed to move to a single prey.

The fish has an omnidirectional field of prey sensitivity [12] (Figure 4A) because of the broad distribution of sensors and electric field described above. This volume is relatively uniform, although there are significant non-uniformities in electric field strength and sensory receptor density [12]. As shown in Figure 4A, as the fish increases its body pitch, the amount of space that it scans while moving increases. The volume the fish can sense prey within while moving is the product of the frontal area of the sensorium (the area that results from projecting the volume to a plane at right angles to the direction of motion), and the distance traveled. For a cuboidal idealization of the complex natural shape of the sensory volume (see Materials and Methods), we found that the projected frontal area increased with body pitch up to a maximum at a body pitch of 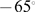 (Figure 5A). At neutral body pitch, the frontal area was 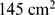, going up by 190% to 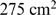 at 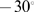 and up by 235% at 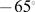.

### Energy Needed to Encounter One Prey

Our energetics model estimates the amount of energy needed to overcome drag forces for the fish to swim to a single prey of the kind used in quantifying the size and shape of the sensorium, *Daphnia magna*. These prey are typically found in stomach content analyses of *Apteronotus albifrons* [17]–[19] and have known energy content (Table 1). We assume that prey are uniformly distributed at the density shown in Table 1.

**Table 1:** 

Parameter and Symbol	Value	Source
Sensory volume cuboid height, *H*	12.5×10^−2^ m	[12] 1
Sensory volume cuboid length, *L*	26.9×10^−2^ m	[12] 1
Sensory volume cuboid width, *W*	11.6×10^−2^ m	[12] 1
Energy content range per prey, *E*_daph_	1–2 J	[26], [45] 2
Prey density, *D*_prey_	5×10^3^m^−3^	[27], [28] 3
Thrust power, *P*_thrust_	0.3×10^−3^ W	Present study
Fish length, *f_l_*	19×10^−2^ m	Present study
Fish mass, *f_m_*	23 g	Present study

1 Sensory volume dimensions scaled by body length (14.4 cm in [12]; 19.0 cm in the present study).

2 Computed from dry mass range of 0.05–0.1 mg per *Daphnia* from [45] and dry weight energy density of 21 J/mg for *Daphnia* quoted in [26].

3 Density is total zooplankton density quoted for South American black water rivers, typical of the kind where *Apteronotus albifrons* is found.

As derived below in Materials and Methods, the equation for estimating the energy in joules needed to overcome drag to reach a single prey is
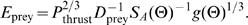
(1)



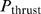
 is the power needed to overcome drag at the reference velocity (during steady state swimming, thrust power must be equal to the power needed to overcome drag). We fixed 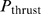 to the power needed to overcome the experimentally measured tow drag at 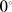 pitch and 15 cm/s, which was 0.3 mW (15 cm/s times the drag force at this velocity, 2 mN, Figure 3). 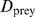 is the density of prey (see Table 1). 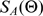 is a function of body pitch angle which returns the area of the sensorium projected to a plane perpendicular to the path of motion. 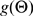 is a function of body pitch such that the drag force is equal to 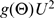, where 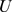 is the velocity of the fish.

As shown in Figure 5B for the curve labeled “2.2 (natural)” the energy needed to encounter one prey at neutral pitch was slightly over 25 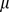
J, going down by around 40% to near 15 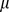
J at the optimal pitch of just over 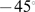, with a similar value at a pitch of 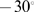.

### How Propulsion is Affected by Body Pitch

**Figure 6:**
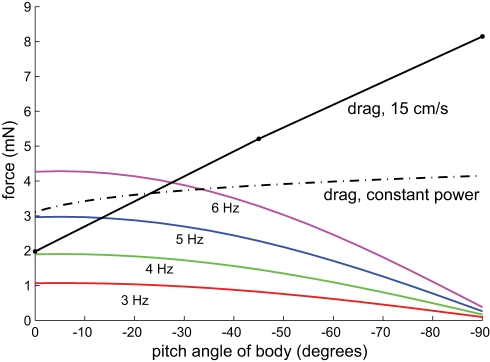
Net propulsive force from fin calculated from Eq. 5 across a set of ribbon fin undulation frequencies versus body pitch angle, compared to drag force. In order to be free swimming at constant velocity, the generated thrust must equal drag. Dash-dotted line shows the estimated drag on the body using the equation 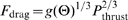 where 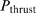 is the total power needed to overcome measured drag at 15 cm/s and zero pitch angle for the fish cast (0.3 mW), and the velocity of the fish is allowed to vary (see Materials and Methods).

Changing the pitch of the body not only affects the drag on the body, and the search rate, it also affects propulsion. The black ghost knifefish generates force by undulating the extended ribbon fin along its underside (Figure 1A) while keeping its body semirigid except for bends to turn left or right [20], [21]. The fin undulations are approximately sinusoidal and travel from one end of the fin to the other—from head to tail for forward movement. The fin generates two different forces: one along the length of the fin (called surge), and one smaller force perpendicular to the fin, pushing the body up (called heave) [21]. As the fin tilts, the forward propulsive force reaches a maximum when the fin base is at an angle of approximately 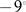 to the horizontal. This is its angle when the body axis is horizontal (e.g., when 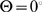, then the fin base is at angle 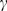 in Figure 1B, approximately 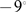). As the fin base tilts past 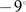 (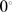 body pitch), the sum of the surge and heave forces projected to the forward direction decreases. This effect is shown by Figure 6, which depicts a family of curves relating forward propulsive force to body pitch (Figure 6). For the purposes of this illustration, we assume that the fish varies its frequency of undulation to vary propulsive force. This appears to be true [20].

### How Sensorium Elongation Affects Energy

We examined the influence of sensorium shape on the energy needed to encounter prey. We define the “elongation factor” as the ratio of the length to height of the sensorium. The effect of elongation factor on projected sensorium area and energy to encounter one prey is shown in Figure 5. The naturally observed elongation factor is 2.2. When the elongation factor was 1.0 (sensorium length equal to height), the energy needed per prey decreased negligibly at low angles before increasing with body pitch angle; essentially, there was no improvement in performance with pitching the body. When the elongation factor was 4.0 (sensorium length four times its height), the energy needed decreased with body pitch angle up to pitch angles of 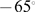. With this elongation factor, the energy needed per prey encounter was typically less than half the energy per prey encounter for the 2.2 elongation factor at relevant body pitch angles. Sensorium elongation makes body pitching progressively more advantageous.

**Figure 7:**
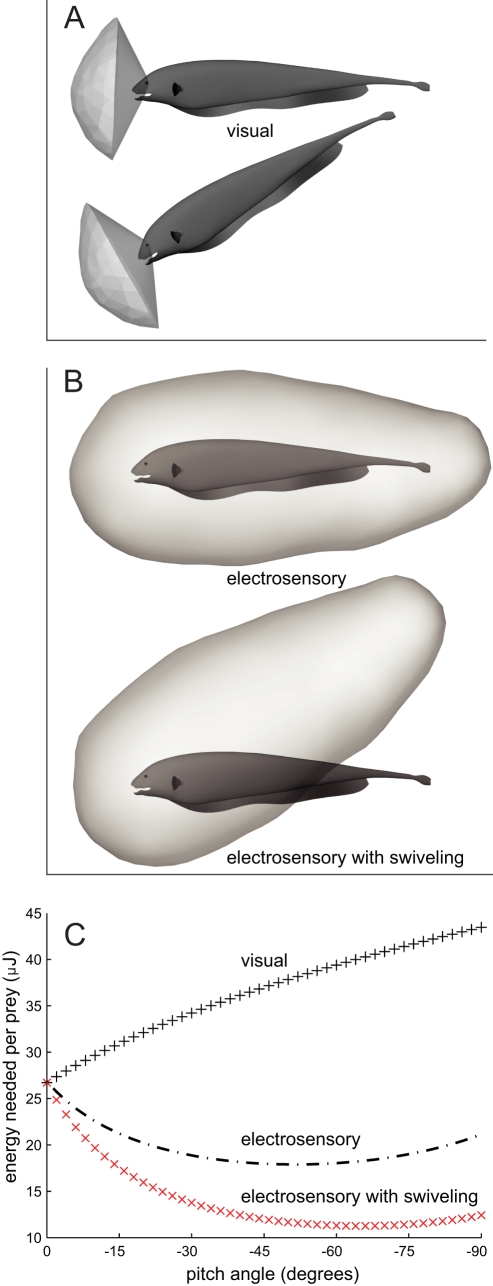
Two hypothetical scenarios: an electric fish hunting with a visual sensorium and with a movable sensorium. (A) An electric fish with vision-like sensorium. A sector of the normal omnidirectional sensorium has been cut to simulate the situation of a sensorium with a similar initial projected area as the normal omnidirectional case, but mediated by vision. In this scenario, we are keeping the sensorium body-fixed. (B) An electric fish with a movable sensorium. In this scenario, the fish is able to swivel the sensorium in pitch, around an axis between the eyes, without pitching the body, similar to the effect of moving eyes in a visual animal. (C) Energetic consequences of the hypothetical sensorium geometries. The solid line is the original case, from Figure 5B. The ‘+’ curve shows the simulation of the effect of a visual sensorium. The ‘x’ curve shows the result of allowing the sensorium to swivel up from the tail while not changing the pitch of the body.

The effect of blunt versus elongated sensoria was further explored through a scenario in which the black ghost has a frontally-directed visual sensorium (see Figure 7A) rather than its normal omnidirectional sensorium (Figure 4A). A fish called the stone moroko (*Pseudorasbora parva*) is a visual predator whose vision-based sensorium for *Daphnia* has been measured ([22]) and is shown in Figure 4B. A cuboidal approximation of the stone moroko visual sensorium is 11.9 cm high (vertical)×12.0 cm long (distance of leading edge from the eyes)×18.7 cm wide (left-right extent). The elongation factor, length over height, is therefore close to 1.0. Given this aspect ratio, there is only a very slight increase in the swept volume of the sensorium with swiveling of the volume in pitch (see the “1.0 (blunt)” curve in Figure 5A). As shown in Figure 4B, this cuboidal approximation overestimates the effect of pitching the conical visual sensorium of the stone moroko.

We will simplify the analysis slightly by 1) making the idealization that projected area does not change with pitch angle because of the aspect ratio of the visual sensorium, and by 2) allowing the projected area of the electrosensory sensorium at 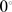, 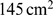 (Figure 5), stand for the projected area of the visual sensorium at 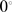, which is 11.9 cm
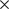
18.7 cm or 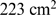. This facilitates comparison to the electrosensory case.

The energetic consequence of this visual sensorium is then obtained by clamping the projected area (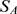) term of the 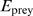 equation to its value when the body is pitched at 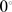, as shown in Figure 7C by the black ‘+’ curve. The energy to overcome drag monotonically increases; the benefit of holding the body at a pitch is lost.

### How Sensorium Mobility Affects Energy

Long range sensing organs, such as eyes and pinna, are often clustered and invested with muscles that enable them to rotate, which in turn rotates their associated sensorium. What effect does sensorium mobility have on the amount of energy needed to encounter prey? In another hypothetical scenario, we examined the consequences of the fish being able to pitch its sensorium around its head without moving its body, illustrated in Figure 7B. We do this by clamping the drag force (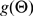) term of the equation for 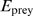 above to its value at 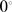, with the result shown in Figure 7C by the red ‘x’ curve. There is a substantial decrease in energy needed per prey. Whereas this sensorium mobility is not biologically possible due to the near-field and broadly distributed nature of electrosense, this example serves to illustrate how sensorium mobility for a far-field sensory system can have beneficial consequences.

## Discussion

### Energy Constraints on Active Sensing Systems

Given the limited availability of energy, all animals must balance the energy load of sensory and neuronal systems with motor and other body systems. However, active sensing animals such as bats, dolphins, and electric fish, have a particularly stringent constraint: they must generate the energy required to perceive their world. Both emitted energy and energy reflected from objects falls as 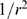 [23], so that the total power attenuation is inversely proportional to 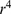. By this, a doubling of sensory range takes sixteen times more energy.

As an example of how constraining the physics of active sensing energy attenuation is, we consider the power the electric fish has to emit to detect prey. The electric fish's self-generated electric field allows them to detect prey at less than a body length away from the body [16]. The energetic cost of electric signal generation was recently measured at 3–22% (depending on time of day and gender) of the total metabolic rate [24]. For a 350 J/day total energy budget for the black ghost knifefish [25], this amounts to a peak of up to about 80 J/day. This power level enables them to detect prey at up to 3 cm [16]. To detect prey at twice this distance, or 6 cm, would require 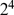, or 16 times more energy, or 1,280 J—four times the total energy budget of the fish. Although the signal generation power measurements used here are for a different species of South American weakly electric fish, the argument is hardly affected even at an order of magnitude lower power.

Given these simple estimates, while all animals have to contend with trade-offs between more energy devoted to sensory systems versus other systems, we can expect these trade-offs to be especially clear in active sensing animals such as electric fish.

### Drag on Body is Offset by Increased Search Rate

**Figure 8:**
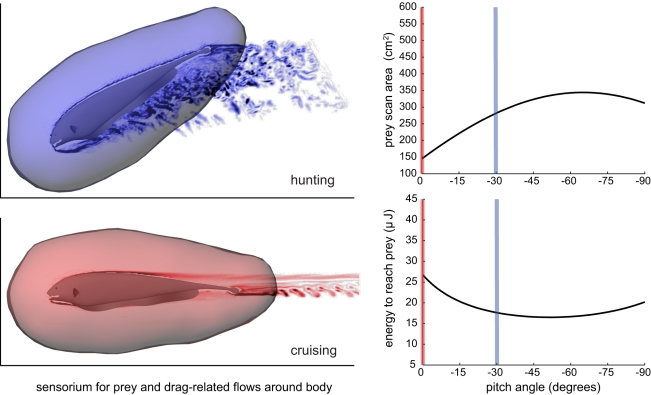
A black ghost with its sensorium for prey, showing computed flow patterns resulting from two different pitch angles. As shown by the high degree of flow separation behind the fish pitched at 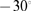 (the orientation it hunts prey in), compared to the laminar flow behind the fish at 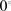, there's significant energy costs associated with angling the body downward due to drag. However, the area scanned for prey by its sensorium while swimming forward increases, as shown by the plot at right. The net effect is that the fish needs less energy to get to its next prey when its body is pitched at 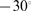. While pitch angles of around 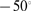 are best in terms of reducing the energy to get to the next prey, this does not incorporate the diminishing propulsive effectiveness of the ribbon fin as the body pitches more. Propulsion drops by about 25% at 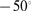 (Figure 6). Because of this effect, the best angle for the fish to swim at will be less than 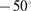.

Figure 8 shows one of our key results in summary form. We have found that as the body pitch increases from zero to 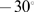, the drag force increases by a factor of between 2–4 times at a search swimming velocity of 15 cm/s. However, this increase in pitch angle also results in a near doubling in the search rate as quantified by projected area of the sensorium. In the simplified model, the balance of these two factors, which is quantified by the energy required to reach one prey (Figure 5B), results in a best pitch angle of around 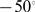. This results in a 40% energy saving over swimming at 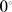. Put another way, the number of prey encountered over a given distance of movement will be nearly doubled due to the near doubling of projected sensorium area.

The measured fish pitch angle during search was 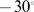 [16], significantly different from the optimum found here. There is an additional factor which will have the effect of reducing this disparity. This is a reduction in propulsive force from the ribbon fin with increased pitch angle, as shown in Figure 6. In this figure, each solid curve shows how the thrust from the fin, with a traveling wave at the indicated frequency, decreases as the body pitches. Across the different undulation frequencies, the propulsive effectiveness of the fin drops around 25% at 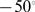. If this effect were to be fully incorporated, the optimal swimming angle would clearly be less than 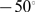.

To illustrate this relationship, consider the dashed line of Figure 6, which shows the drag force on the body when total power expended for swimming (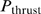) is clamped at a specific value, calculated given the drag force (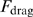) at a pitch angle of 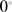 and a velocity (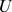) of 15 cm/s (0.2 mN
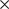
15 cm/s, which is 0.3 mW). Given that 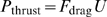, an increase in drag requires a decrease in velocity to keep power fixed, resulting in a lower velocity as drag increases with pitch angle. Therefore, the dashed line indicates the thrust needed to overcome this drag when the power available is the same as when the fish swims horizontally. The intersection between the dashed line and the thrust curve indicates the approximate pitch angle required to move with a constant velocity, as propulsive thrust balances body drag resulting in zero acceleration. In particular, at the highest undulation frequency shown, 6 Hz, the fish would need to swim at 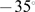, significantly below the 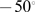 pitch that would be best if loss of thrust with increased pitch were not a consideration.

### Limitations of the Energy Estimates

While we have found that the mechanical energy needed to find each prey is on the order of tens of microjoules, a small fraction of the energy gained per prey (on the order of a joule; see Table 1), the mechanical energy expended to overcome drag is only a fraction of the total energy the animal will use in finding each prey. This is because 1) not all the energy in food is converted to available energy [26]; 2) not all the available energy is used for swimming muscles (e.g., we estimate the mechanical power used for swimming at 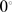 pitch and 15 cm/s is 0.3 mW (the velocity times the drag at this velocity, 15 cm/s
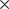
2 mN), while metabolic rate is on the order of 0.4 mW [25]); and 3) not all the energy used for swimming muscles is converted into thrust. These factors combined are around a factor of ten. There is also significant uncertainty in the prey density numbers. The energy needed per prey will double if the prey density is half that used for these estimates (5,000 prey per cubic meter). The density appears to vary between 1,000–5,000 individuals per cubic meter for rivers typically inhabited by Amazonian electric fish [27], [28]. However, this includes many different insect species and it is unclear what fraction of these are prey the fish would eat. Despite these uncertainties, the fish has few ways at its disposal for increasing search rate at a given velocity beyond changing pitch angle. For example, it cannot increase its sensorium size because it does not vary its electric field strength, although another species of weakly electric fish has recently been shown to vary its electric field strength [29]. Thus the increased mechanical load on the fish with increased body pitch is an appropriate variable to examine.

### The Effect of Sensorium Shape

A key factor in the beneficial effect of pitching the body is the shape of the sensorium. More specifically, how the projected area of the sensorium changes as a function of the sensorium position control variable, in this case body pitch (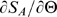), is crucial. As the sensorium becomes less elongated, the increase in projected area with increased pitch angle becomes negligible, and thus the benefit of body pitching disappears. This is shown in Figure 5A and B. As the sensorium becomes more elongated (elongation factor 4.0), the projected area increases more rapidly with pitch angle, and the net energy needed per prey decreases more rapidly. The opposite holds for the cube-like sensorium (elongation factor 1.0): there is nearly no increase in projected area with pitch angle, and thus the energy needed per prey only increases with pitch angle due to increased drag forces.

As another way to examine this effect, we computed the energetic consequences of the black ghost using a visual sensorium, illustrated in Figure 7A. The visual sensorium for the detection of the same type of prey used in this study, *Daphnia*, in a visual predatory fish (the stone moroko) has been measured to be 11.9 cm high (vertical)×12.0 cm long (distance from eye to leading edge)×18.7 cm wide (left-right) (Figure 4B). This sensorium has an elongation factor (length to height) of unity, so the projected area changes little with rotation in pitch. As a visually-guided animal with movable eyes, the stone moroko can choose to rotate its eyes with its oblique muscles to control the pitch angle of its sensorium [30]. For the purpose of this example, let's facilitate the comparison to the elongated body-fixed sensorium of the black ghost by supposing that this artificial visual sensorium is also body-fixed, as depicted in Figure 7A. Thus, the fish changes the pitch of its body to change the pitch of the sensorium. The effect of this faux visual sensorium on energy is shown in Figure 7C. There is no benefit to pitching when the effect of the elongated sensorium is removed, and only the cost of overcoming drag remains for the artificial case of a body-fixed visual sensorium.

These results indicate that an elongated sensorium is beneficial. In this particular group of species, an elongated sensorium goes along with an elongated body that is characteristic of the knifefish body plan, common across some 180 different species (Gymnotidae) [31], and the distributed nature of the electrosensory system of these fishes.

For the stone moroko, a fish which swims by “tail-wagging” (the carangiform mode), the instability in yaw induced by tail beating results in high yaw maneuverability [1], and would facilitate prey capture lateral to the fish body. In addition, left-right eye movements will sweep the sensorium in azimuth. Therefore, this fish's vertically flattened sensorium, over one-and-a-half times wider than it is tall, seems likely to be beneficial. Further amplifying this point, Figure 4B shows that pitching the sensorium would in fact decrease the swept volume slightly. The relevant elongation factor for this fish will be length to width, since height will only have a constant factor effect on how projected area changes with azimuthal angle.

### Decoupling Sensorium Movement from Body Movement

Weakly electric fish have a body-fixed sensorium. If it were at all possible to change the position of the sensorium without changing body position, as animals that rotate their eyes or turn their heads can [9], one possible scenario would allow the animal to have all of the sensory advantages of pitching the body, with none of the drag costs. In this scenario, imagine the fish could tilt the back of its sensorium up as illustrated in Figure 7B, but without tilting the body—analogous to how some animals can rotate their visual sensorium without moving their bodies. We can assess the energy implications of this scenario through the use of Eq. 2, by fixing the drag force (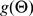) to its value when the body is at 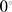 pitch, while allowing 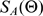 to vary. The result is shown by the ‘x’ curve of Figure 5B. Being able to dynamically reposition the sensorium without moving the body results in more than a factor of two decrease in energy per prey at 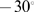, and even more at larger angles.

Decoupling sensorium movement from whole body movement has been an ancient theme of vision, our most powerful teleceptive sensory modality. Independent eye movement and stabilization goes back to the very first vertebrates [32]. There are many benefits to eye movements, such as minimization of motion blur due to self movement and movement of the object of fixation [9], but clearly not having to reposition the body to see something initially out of view can economize on energy [30]. Given that body mass is considerably larger than sensory organ mass, it also saves on time. One cost, however, is the need to translate the coordinates of perceptual information arriving in sensory-organ-fixed coordinates to the coordinates of the body, demanding significant neuronal processing. The tectum, or superior colliculus, is one structure where this occurs (review: [33]).

Whereas eye movement is quite ancient, the ability to turn the head is relatively recent in vertebrates. Our earliest evidence of this ability is from a 375 million year old fossil of an animal that appears to be a transitional form between fish and tetrapods, *Tiktaalik roseae* [34]. Some active sensing animals exploit head movements for sweeping their sensoria horizontally and vertically while keeping their body on a fixed course. For example, bats nearly double the angular range of their sonar-based sensorium by combining pinna and head movements [35]–[37], and dolphins have also been shown to use head movements to manipulate their sonar-based sensorium to a similar end [38]. Rats also exploit this freedom, combining head movements with whisker movements to palpate objects [39].

Having relatively light and independently movable sensory appendages is a ubiquitous feature of animal body plans. It is particularly powerful for teleceptive systems such as vision and audition. The analysis here highlights how advantageous it can be to decouple sensorium movement from whole body movement from an energetics standpoint. It may also suggest that when developing assistive technologies for people with sensory challenges, a sensorium whose movement is independently controllable from body movement can be particularly helpful.

### Conclusion

Although there is a significant literature of how mechanical considerations enter into sensory performance in a large number of systems, and a growing literature on the metabolic cost of information, there has been little examination of how these two domains overlap and trade-off with one another. While measures of performance in these two areas typically are not commensurable, the impact of a change in sensing or movement on the net energy balance of an animal provides a basis of comparison. We have been able to quantify how this animal trades-off movement efficiency for sensory performance in prey search behavior. A simplified model illuminates why the animal searches with its body in a drag-inducing position, and suggests a possible basis for why this group of animals has evolved an unusual degree of elongation in their body plan. This model also illustrates the benefits of sensorium mobility that is decoupled from whole-body movement.

In the traditional view, the nervous system performs the computational “heavy lifting” in an organism. This view neglects, however, the critical role of morphology, biomaterials, passive mechanical physics, and other pre-neuronal or non-neuronal systems. Given that neurons consume forty times more energy per unit mass than structural materials such as bone [40], and twenty times as much as muscle ([5], after [6]), there are clearly advantages to distributing tasks between these tissues in a way that improves energetic efficiency. In this “bone-brain continuum” view [41], animal intelligence and behavioral control systems can only be understood using integrative modeling approaches that expose the computational roles of both neural and non-neural substrates and their close coupling in behavioral output. The infomechanical approach taken here, in which information and mechanics are jointly examined with regard to energy consequences, is one such approach that can facilitate a more integrative understanding of animal system design.

## Materials and Methods

### Empirical Drag Measurements

An accurate urethane cast of a 190 mm long *Apteronotus albifrons* made for a prior study [42] was bolted to a rigid rod. This was suspended from a custom force balance that used three miniature beam load cells (MB-5-89, Interface Inc., Scottsdale AZ USA). For force balance and calibration details, see [43]. The fish cast was towed through a large tank that was 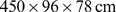 in length, width, and depth (GALCIT towtank, Caltech) using a gantry system driven by a speed-controlled DC servomotor above the tank [43]. Trials were conducted at three speeds: 10, 12, and 15 cm/s, and three angles to the flow: 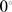, 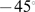, and 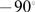. Only the data collected after the startup force transient had settled was analyzed, until just before the end of the towing distance (300 cm). The data was filtered with a digital Butterworth low pass filter (cutoff at 5 Hz) to remove transducer transients prior to further statistical analysis.

### Computational Drag Estimates

We used a custom computational fluid dynamics solver to obtain the drag force on a fish model at different towing velocities. The fish model was derived from the same urethane cast as was used for the tow-tank measurements [42]. It is assumed to be rigid. In the numerical simulations, it is towed at 10, 15, and 20 cm/s, and three angles to the flow: 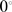, 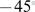, and 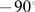. All of the simulations were performed using the San Diego Supercomputer Center's IA-64 Linux Cluster, which has 262 compute nodes each consisting of two 1.5GHz Intel Itanium 2 processors running SuSE Linux. The computational fluid dynamics code was written in Fortran 90 and C (for details, see [21]).

### Projected Area of Sensorium

**Figure 9:**
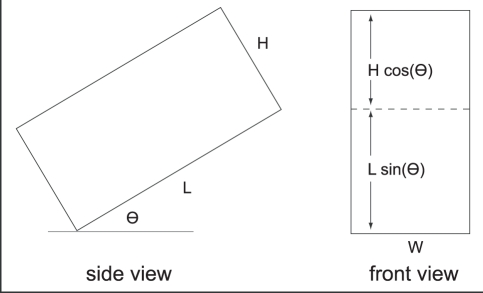
Schematic showing simplified model with cuboidal sensorium. 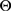
 is the pitch angle of the body.

In a prior study we used a combination of empirical measurements and computational models to determine the 3D volume around the fish body where a typical prey item, *Daphnia magna*, could be detected (Figure 4A) [12]. We idealized the resulting electrosensory sensorium as a cuboid (Figure 9) whose width, height, and length is matched to the maximal dimensions of this volume, after scaling for body size (the body length for the [12] study was 14.4 cm, while it is 19.0 cm in this study). The resulting dimensions are shown in Table 1.

As shown by Figure 9, the projected area of this cuboidal sensorium in the direction of travel (its silhouette if you were to look at it directly along the path of its approach) is simply 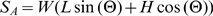. We varied the ratio of the length to the height of the cuboidal sensorium. To assess the impact of elongation of this volume on projected area with pitch angle 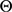, we varied the ratio of the length to the height of the cuboidal sensorium (the elongation factor). These two dimensions were chosen because by the above equation for 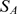, varying the width only results in a constant factor change in the projected area with respect to 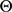. The naturally observed elongation factor was 2.2.

### Energetics Model

We assume that prey are uniformly distributed at the density shown in Table 1. As shown in Figure 9, the projected search area is 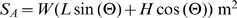. Thus, the total water volume scanned for prey when the fish moves distance 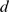 will be 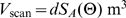. The number of prey detected in that volume will be the volume times the prey density, or 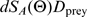. The distance travelled to get one prey will then be 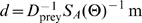.

We fit our measured drag data to a function of the form 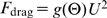, where 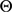 is in degrees. The result is 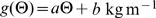, where 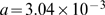 and 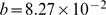, with an 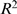 of 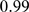. Thus thrust power 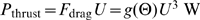. We can rearrange this to solve for 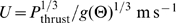.

We rearrange 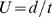 to solve for 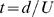 and use the solution for 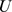 from above to solve for 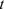, the time required to find one prey. Then we multiply this by 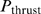 to solve for the energy expended to overcome drag in obtaining one prey:
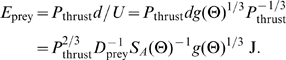
(2)


For 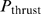, we used the power needed to overcome the experimentally measured tow drag at 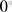 pitch and 15 cm/s, which was 0.3 mW (15 cm/s
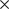
2 mN) (Figure 3).

### Computational Thrust Force Estimates

For a previous study we used a computational model of a non-translating, non-rotating fin deforming in a sinusoidal pattern with time [21]. The instantaneous velocity of each point on the fin is specified as a function of time. The no-slip and no-penetration boundary conditions are imposed on the surface of the fin using an immersed boundary formulation, and the fluid flow around it is fully resolved using finite difference methods of 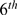 order in space and 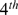 order in time. The complete details of the computational algorithm and method are given in [21], [44].

Mean forces on the fin were calculated as the time average of the hydrodynamic forces on the fin over at least one period of oscillation, after a quasi-steady state is reached. As shown in [21], the force in newtons from the fin followed the correlation
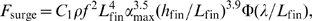
(3)where 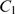 is a constant equal to 86.03, 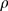 is the density of water (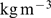), 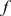 is the frequency of the traveling wave on the fin (Hz), 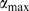 is the maximal angular excursion of the traveling wave (radians), 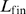 is the fin length (m), 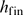 is the height of the fin (m), 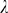 is the wavelength of the traveling wave (m), and 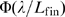 is a function of the specific wavelength which can be approximated by:
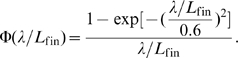
(4)


This equation estimates the propulsive force parallel to the fin, or surge force. However, in addition to this force, the fin also generates a small force that is perpendicular to the fin base, pushing the body upward. This force, termed heave, has a magnitude of about 25% of the surge force for typical motion patterns [21]. Because of the relative magnitudes of the surge and heave forces, the angle of the fin that would maximize forward thrust is 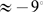. This angle is nearly identical to the observed fin insertion angle on the body (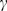 in Figure 1B) when the fish is swimming straight. By knowing the surge force, and this angle, we can therefore compute the heave force as the tangent of the fin base angle times the surge force. As the body pitches, the contribution of the parallel surge force to thrust will vary with the cosine of the sum of the body pitch angle 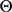 and fin base angle 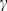, whereas the contribution of the normal heave force will vary with the sine of the sum of these two angles. Thus the net force will be:
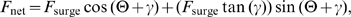
(5)where 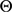 is the body pitch angle, and 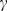 is the angle of the fin base with respect to the body axis at 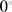 body pitch (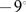; shown in Figure 1B). For these force estimates, we used the length of the fin of the fish used for drag estimates (12.7 cm), a fin height of 1 cm, and typically observed kinematic values of an 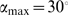, and two waves along the fin (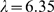 cm) [20], [21].

To compare thrust to drag when the power expended on swimming is fixed, we derive the relationship between the drag function 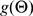 and swimming power. Based on 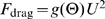 and 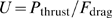, 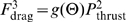. Thus 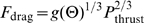.

### Effect of Sensorium Shape

To assess the effect of sensorium shape, we examined elongation factors of 1.0 and 4.0 by changing the sensorium length to be equal to its normal height, and four times its normal height, respectively. We then examined the energetic consequences of these sensorium morphologies. This was done through the equation describing the energy needed per prey encounter described below (Equation 2) through changing the function (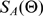) that returns projected sensorium area given the pitch of the body.

For the artificial elongation factors of 1.0 and 4.0, we make the following simplification. A change in elongation factor normally would be accompanied by a change in body elongation. This is because the electric organ and sensors, which together form the sensorium [12], are along the full length of the fish; therefore a change in relative length of the sensorium would necessitate a change in body length. Any change in body length would in turn affect the drag force on the body and thus the energy needed per prey through the 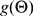 term of Equation 2. Although this was not considered here due to the extensive computational demands of the drag study, the results of simple sensitivity analyses suggest that this simplification has negligible effect on the qualitative trends.

### Energetics of Fixed Sensorium Area and Sensorium Mobility

We examined the energetic consequences of two “what if” scenarios: 1) There is no increase in projected sensorium area as the body pitches. To do this, we clamp 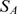 to its value at 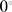. 2) There is no increase in body drag as the body pitches. This would be the case if the fish were able to independently control the pitch angle of its sensorium, analogous to how animals with movable eyes or heads can change the position of their visual sensory volume without changing body position. To do this, we clamp the drag term 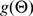 to its value at 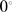.

## References

[1] Weihs D (2002). Stability versus maneuverability in aquatic locomotion.. Integr Comp Biol.

[2] Harris JE (1936). The role of the fins in the equilibrium of the swimming fish. I. Wind-tunnel tests on a model of *Mustelus canis* (Mitchill).. J Exp Biol.

[3] Webb PW (1984). Form and function in fish swimming.. Sci Am.

[4] Alexander RM (1999). Energy for animal life. Oxford animal biology series.

[5] Aschoff J, Günther B, Kramer K (1971). Energiehaushalt und Temperaturregulation.

[6] Aiello LC, Wheeler P (1995). The expensive-tissue hypothesis: The brain and the digestive system in human and primate evolution.. Curr Anthropol.

[7] Niven JE, Laughlin SB (2008). Energy limitation as a selective pressure on the evolution of sensory systems.. J Exp Biol.

[8] Laughlin SB, Barth FG, Schmid A (2001). The metabolic cost of information-a fundamental factor in visual ecology.. Ecology of Sensing.

[9] Land MF (1999). Motion and vision: why animals move their eyes.. J Comp Physiol A.

[10] Weissburg MJ, James CP, Smee DL, Webster DR (2003). Fluid mechanics produces conflicting constraints during olfactory navigation of blue crabs, *Callinectes sapidus*.. J Exp Biol.

[11] Keller TA, Powell I, Weissburg MJ (2003). Role of olfactory appendages in chemically mediated orientation of blue crabs.. Mar Ecol-Prog Ser.

[12] Snyder JB, Nelson ME, Burdick JW, MacIver MA (2007). Omnidirectional sensory and motor volumes in an electric fish.. PLoS Biology.

[13] Nelson ME, MacIver MA (1999). Prey capture in the weakly electric fish *Apteronotus albifrons*: Sensory acquisition strategies and electrosensory consequences.. J Exp Biol.

[14] Turner RW, Maler L, Burrows M (1999). Special issue on electroreception and electrocommunication.. J Exp Biol.

[15] Bullock TH, Hopkins CD, Popper AN, Fay RR (2005). Electroreception (Springer Handbook of Auditory Research).

[16] MacIver MA, Sharabash NM, Nelson ME (2001). Prey-capture behavior in gymnotid electric fish: Motion analysis and effects of water conductivity.. J Exp Biol.

[17] Marrero C (1987). Notas preliminares acerca de la historia natural de los peces del bajo llano. I. Comparación de los hábitos alimentarios de tres especies de peces Gymnotiformes, en el Río Apure (Edo Apure), Venezuela.. Rev Hydrobiol Trop.

[18] Winemiller KO, Adite A (1997). Convergent evolution of weakly electric fishes from floodplain habitats in Africa and South America.. Env Biol Fishes.

[19] Mérigoux S, Ponton D (1998). Body shape, diet and ontogenetic diet shifts in young fish of the Sinnamary River, French Guiana, South America.. J Fish Biol.

[20] Blake RW (1983). Swimming in the electric eels and knifefishes.. Can J Zool.

[21] Shirgaonkar AA, Curet OM, Patankar NA, MacIver MA (2008). The hydrodynamics of ribbon-fin propulsion during impulsive motion.. J Exp Biol.

[22] Asaeda T, Park BK, Manatunge J (2002). Characteristics of reaction field and the reactive distance of a planktivore, *Pseudorasbora parva* (Cyprinidae), in various environmental conditions.. Hydrobiologia.

[23] Nelson ME, MacIver MA (2006). Sensory acquisition in active sensing systems.. J Comp Physiol A.

[24] Salazar VL, Stoddard PK (2008). Sex differences in energetic costs explain sexual dimorphism in the circadian rhythm modulation of the electrocommunication signal of the gymnotiform fish *Brachyhypopomus pinnicaudatus*.. J Exp Biol.

[25] Julian D, Crampton WGR, Wohlgemuth SE, Albert JS (2003). Oxygen consumption in weakly electric neotropical fishes.. Oecologia.

[26] Mittelbach GG (1981). Foraging efficiency and body size: A study of optimal diet and habitat use by bluegills.. Ecology.

[27] Saunders JF, Lewis WM (1988). Zooplankton abundance in the Caura River, Venezuela.. Biotropica.

[28] Saunders JF, Lewis WM (1988). Zooplankton abundance and transport in a tropical white-water river.. Hydrobiologia.

[29] Markham MR, McAnelly ML, Stoddard PK, Zakon HH (2009). Circadian and social cues regulate ion channel trafficking.. PLoS Biology.

[30] Walls GL (1962). The evolutionary history of eye movements.. Vision Res.

[31] Albert JS, Crampton WGR (2005). Diversity and phylogeny of neotropical electric fishes (Gymnotiformes).. Electroreception.

[32] Fritzsch B (1998). Evolution of the vestibulo-ocular system.. Otolaryngol Head Neck Surg.

[33] Masino T (1992). Brain-stem control of orienting movements - intrinsic coordinate systems and underlying circuitry.. Brain Behav Evol.

[34] Daeschler EB, Shubin NH, Jenkins FA (2006). A Devonian tetrapod-like fish and the evolution of the tetrapod body plan.. Nature.

[35] Kalko EK (1995). Insect pursuit, prey capture and echolocation in *Pipistrelle* bats (Microchiroptera).. Anim Behav.

[36] Henze D, Oneill WE (1991). The emission pattern of vocalizations and directionality of the sonar system in the echolocating bat, *Pteronotus parnelli*.. J Acoust Soc Am.

[37] Ghose K, Moss CF (2003). The sonar beam pattern of a flying bat as it tracks tethered insects.. J Acoust Soc Am.

[38] Herzing DL, Thomas JA, Moss CF, Vater M (2004). Social and nonsocial uses of echolocation in free-ranging *Stenella frontalis* and *Tursiops truncatus*.. Echolocation in Bats and Dolphins.

[39] Sellien H, Eshenroder D, Ebner F (2005). Comparison of bilateral whisker movement in freely exploring and head-fixed adult rats.. Somatosens Motor Res.

[40] Martin AW, Fuhrman FA (1955). The relationship between summated tissue respiration and metabolic rate in the mouse and the dog.. Physiol Zool.

[41] MacIver MA, Robbins P, Aydede M (2009). Neuroethology: From morphological computation to planning.. The Cambridge Handbook of Situated Cognition.

[42] MacIver MA, Nelson ME (2000). Body modeling and model-based tracking for neuroethology.. J Neurosci Meth.

[43] Ringuette M, Milano M, Gharib M (2007). Role of the tip vortex in the force generation of low-aspect-ratio normal flat plates.. J Fluid Mech.

[44] Shirgaonkar AA, MacIver MA, Patankar NA (2009). A new mathematical formulation and fast algorithm for fully resolved simulation of self-propulsion.. J Comput Phys.

[45] Ward TJ, Robinson WE (2005). Evolution of cadmium resistance in *Daphnia magna*.. Environ Toxicol Chem.

